# Acceptance of Technologies for Aging in Place: A Conceptual Model

**DOI:** 10.2196/22613

**Published:** 2021-03-31

**Authors:** Christina Jaschinski, Somaya Ben Allouch, Oscar Peters, Ricardo Cachucho, Jan A G M van Dijk

**Affiliations:** 1 Research Group Technology, Health & Care Saxion University of Applied Sciences Enschede Netherlands; 2 Digital Life Amsterdam University of Applied Sciences Amsterdam Netherlands; 3 Saxion University of Applied Sciences Enschede Netherlands; 4 Leiden Institute of Advanced Computer Science (LIACS) Leiden University Leiden Netherlands; 5 University of Twente Enschede Netherlands

**Keywords:** ambient assisted living, assistive technology, healthy aging, technology adoption, theory of planned behavior, structural equation modeling

## Abstract

**Background:**

Older adults want to preserve their health and autonomy and stay in their own home environment for as long as possible. This is also of interest to policy makers who try to cope with growing staff shortages and increasing health care expenses. Ambient assisted living (AAL) technologies can support the desire for independence and aging in place. However, the implementation of these technologies is much slower than expected. This has been attributed to the lack of focus on user acceptance and user needs.

**Objective:**

The aim of this study is to develop a theoretically grounded understanding of the acceptance of AAL technologies among older adults and to compare the relative importance of different acceptance factors.

**Methods:**

A conceptual model of AAL acceptance was developed using the theory of planned behavior as a theoretical starting point. A web-based survey of 1296 older adults was conducted in the Netherlands to validate the theoretical model. Structural equation modeling was used to analyze the hypothesized relationships.

**Results:**

Our conceptual model showed a good fit with the observed data (root mean square error of approximation 0.04; standardized root mean square residual 0.06; comparative fit index 0.93; Tucker-Lewis index 0.92) and explained 69% of the variance in intention to use. All but 2 of the hypothesized paths were significant at the *P*<.001 level. Overall, older adults were relatively open to the idea of using AAL technologies in the future (mean 3.34, SD 0.73).

**Conclusions:**

This study contributes to a more user-centered and theoretically grounded discourse in AAL research. Understanding the underlying behavioral, normative, and control beliefs that contribute to the decision to use or reject AAL technologies helps developers to make informed design decisions based on users’ needs and concerns. These insights on acceptance factors can be valuable for the broader field of eHealth development and implementation.

## Introduction

### Background

Demographic predictions show a growing number of people at risk for age-related chronic diseases and with a potential need for long-term care. At the same time, there is a growing shortage of caregivers. With the pressing demand for care, the workload for formal and informal caregivers is steadily increasing, negatively affecting their physical and mental well-being [1,2]. These developments put the sustainability of our current health care system at risk [3].

To address these challenges, European care reforms have induced a shift from institutionalized care to more care at home and aging in place. Similarly, the European Union (EU) has embraced an active aging policy strategy that emphasizes good health, security, and participation [4,5]. State-of-the-art assistive technologies, also known as ambient assisted living (AAL) technologies, are viewed as a vital contributor to this strategy.

### Ambient Assisted Living

The term AAL has been introduced by the EU to describe the use of a new generation of information and communication technology (ICT)-based assistive technologies that provide holistic support to older adults in managing their health, remaining independent, and staying involved with their community. AAL technologies are also directed at caregivers to relieve some of their burden and support them in the coordination and management of care tasks [6,7].

AAL builds on the classic principles of ambient intelligence (embedded, context-aware, personalized, adaptive, and anticipatory) [8] to create supportive environments for older adults and their caregivers. AAL is an umbrella term for a range of state-of-the-art technologies such as smart home technology, mobile and wearable technology, and assistive robotics [9]. We previously defined AAL as follows [7]:

State-of-the-art ICT-based solutions that build on the principles of ambient intelligence to create intelligent environments that provide all-encompassing, non-invasive, and pro-active support to older adults and have the ultimate goal to maintain their independence, enhance their overall quality of life, and support their caregivers.

Application areas are broad and include, for example, health monitoring, activity monitoring, medication management, fall detection, reminder and planning systems, interactive games and storytelling, care management, social companion robots, and ambient awareness systems.

Although there are high hopes for AAL technologies to solve the challenges of the aging population, different systematic reviews conclude that the technology readiness level of these applications is still low and that most applications have not yet matured into the implementation phase. In addition, scientific evidence for the effectiveness of these technologies is weak and efficiency outcomes are almost nonexistent [6,10,11]. Furthermore, research in the AAL area is still predominately technology oriented [6,12], and there is little theoretical understanding of the user’s perspective [11,13]. Hence, there is a need for further research on user acceptance.

### The Importance of User Acceptance

User acceptance is key to the successful adoption and diffusion of new technologies. Indeed, several researchers have concluded that understanding user acceptance and incorporating user needs is essential to the successful digitization of the health care sector [14-18]. In the context of AAL, the slow deployment of AAL systems has been attributed to the lack of user acceptance and missing focus on user needs [9,13,19]. Loss of privacy [20-22] and the fear of substituting face-to-face interaction [23-26] are examples of acceptance barriers found in previous research. This is not surprising considering the pervasiveness of these technologies [27]. These applications are designed to be placed in personal environments or directly on the body, collect and store sensitive data, influence behavior and habits, and take over tasks that are usually carried out by the older adults themselves or a human caregiver.

The insufficient understanding of users’ needs is also reflected in ageist stereotypes, which are still common in this field [28-30]. These studies portray older adults as a homogeneous group that is frail and lonely and has low technology literacy. To combat these stereotypes, researchers need to adopt a more user-centered mindset and develop a deeper understanding of the user’s point of view.

Although the number of studies on user acceptance and user needs has slowly increased over the last couple of years, most research still lacks a solid theoretical foundation to explain and underpin their results [11]. This is also confirmed by Blackman et al [31], who concluded that AAL research is rich in data but poor in theory. A solid theoretical foundation is crucial for understanding the underlying social, psychological, and behavioral mechanisms of the acceptance process. A related concern is the lack of large-scale quantitative research on user acceptance in this area [11,13]. More quantitative approaches are needed to understand the relative importance of acceptance factors, identify their underlying relationships, and make statistically grounded and externally valid inferences about their influence on the acceptance process. Developing a stronger theoretical and statistically grounded understanding of user acceptance in AAL research will improve AAL conceptualization and development. At the same time, it will increase the likelihood of future acceptance by intended users.

### The Conceptual Model of AAL Acceptance

Technology acceptance occurs over time and consists of different stages [32-36]. Owing to the overall low maturity of AAL technologies, it was decided to focus on early user acceptance, meaning the factors that contribute to the initial intention to use or reject AAL technology in the future.

Over the years, several theories and models have been developed to explain technology acceptance, including the technology acceptance model (TAM) [37,38], the unified theory of acceptance and use of technology (UTAUT) [39], and the theory of planned behavior (TPB) [40]. Although TAM and UTAUT are popular choices in the field of eHealth [18], we chose TPB as a theoretical foundation for several reasons. First, TPB is a well-known and validated psychological theory to understand and explain human behavior, including technology acceptance [41-44] and health-related behaviors [45,46]. It has also been applied to understand the adoption of assistive devices [47] and eHealth applications [48]. In contrast, UTAUT is an eclectic model that lacks a strong theoretical foundation [49]. Second, TPB provides an ideal basis for understanding early user acceptance by specifically focusing on the attitudinal, social, and normative belief structure that leads to the intention to use a technology. These insights are very informative for further development and implementation of AAL. In contrast, TAM’s predominant focus on usefulness and ease of use provides little valuable insights for the design and implementation of new technologies [50]. Third, TPB is explicitly open to the inclusion of more variables [40] and therefore forms a good starting point for developing a new model of AAL acceptance.

Intention is a central construct in TPB and viewed as an immediate determinant of actual behavior. Intention is defined as an “indication of a person’s readiness to perform a given behavior.” According to TPB, intention is determined by 3 variables: attitude toward the behavior, subjective norm, and perceived behavioral control. Attitude is defined as “the degree to which performance of the behavior is positively or negatively valued.” Subjective norm is defined as the “perceived social pressure to engage or not to engage in a behavior.” Perceived behavioral control can be described as “people's perceptions of their ability to perform a given behavior.” Following an expectancy value approach, in TPB, attitude is determined by a set of behavioral beliefs about the outcome of a given behavior, weighted by the evaluation of that outcome. Subjective norm is determined by a set of normative beliefs concerning the expectations of important referents, weighted by the motivation to comply. Finally, perceived behavioral control is determined by several control beliefs, weighted by its perceived power [40,51,52].

**Figure 1:**
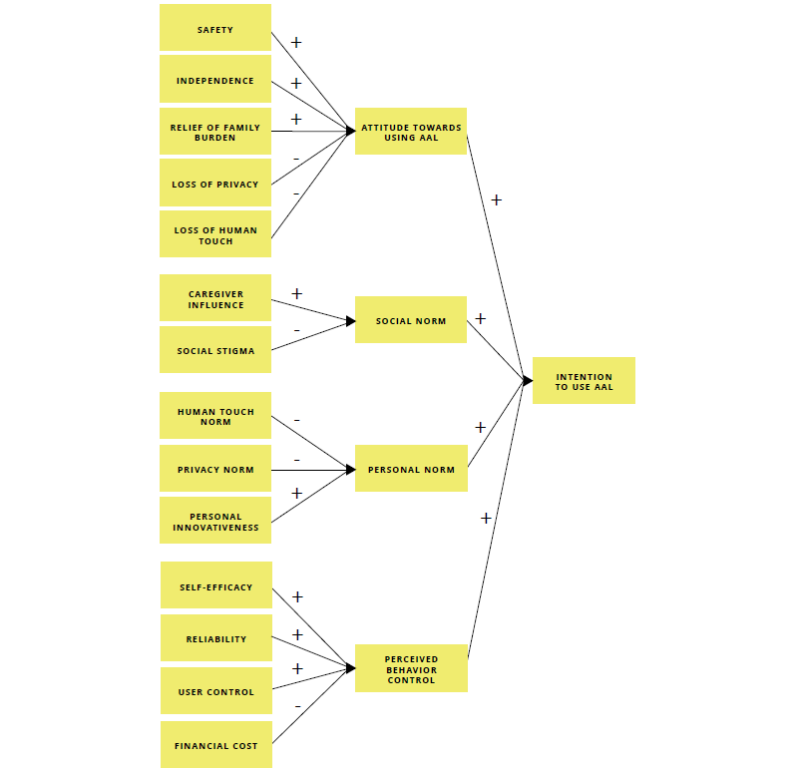
Conceptual model of ambient assisted living acceptance.

In line with TPB, intention to use *AAL* is proposed as the key dependent variable in our conceptual model, with *attitude toward using AAL, social norm, and perceived behavioral* control as direct ascendants (Figure 1). Personal norm was added as an additional predictor of intention, thereby answering to the appeal of previous researchers to consider different normative mechanisms for TPB [45,53]. We define personal norm as “people’s self-based standards or expectations for AAL use that flow from one’s internalized values,” thereby referring to Schwartz [54]. The construct was operationalized in terms of self-identity, drawing on the work of Lee et al [55] and Sparks and Shepherd [56].

For the conceptual model, the underlying behavioral, normative, and control belief structures were decomposed into specific multidimensional belief constructs, as suggested by Taylor and Todd [43]. The advantage of this approach is that it emphasizes the relevant beliefs antecedents for AAL acceptance and, consequently, provides more directive insights for the design of AAL technologies [43]. We drew on earlier user research in the field [23,57-59] and our own qualitative user studies [60,61] to select the relevant underlying beliefs. This *resulted in safety, independent living*, and *relief of family burden* as positive belief antecedents for attitude and *loss of privacy* and *loss of human touch* as negative belief antecedents. *Caregiver influence* was proposed as a positive antecedent of social norm, whereas social stigma was proposed as a negative antecedent. Personal norm was hypothesized to be positively influenced by one’s *personal innovativeness*, whereas human touch norm and privacy norm were suggested as negative antecedents. Finally, perceived behavioral control was hypothesized to be positively influenced by *self-efficacy, reliability*, and level of user control and negatively influenced by financial cost. Multimedia Appendix 1 displays an overview of the underlying belief constructs and their definitions.

## Methods

### Overview

A web-based survey of older adults was conducted in the Netherlands to validate the conceptual model. A Dutch ISO (International Organization for Standardization)–certified research agency was hired to distribute the survey. The agency is an expert in web-based fieldwork and manages a panel of 110,000 members with diverse sociodemographic backgrounds.

### Participants

Older adults aged between 55 and 85 years were specified as the target population to include older adults with different living and work situations, different perceptions of health and quality of life, different support needs, and different levels of technology experience. Our aim is to adequately represent this highly heterogeneous target group [62,63]. The lower boundary of the age requirement was set at 55 years to include the perspective of the future generation of older adults. Predefined age quotas were used to obtain a representative sample.

Upon invitation, 2113 older adults participated in the survey. Of these participants, 679 participants did not complete the survey, most of whom stopped immediately after the introduction page. Another 138 participants were removed from the sample because of: incomplete response patterns, exceptionally short response times, straight lining, and insufficient understanding of the presented AAL material. This led to a response rate of 61.33% (1296/2113) and a total of 1296 cases for further analysis. The final sample was representative of the older Dutch adult population in terms of age (55-64 years: n=555, 42.82%; 65-74 years: n=497, 38.35%; 75-85 years: n=244, 18.83%) and gender (male: n=637, 49.15%; female: n=659, 50.85%) [64]. Most of the participants (n=1227, 94.68%) had no user experience with AAL applications. All other sample characteristics are presented in Table 1.

**Table 1:** Sample characteristics (N=1296).

Variables		Values, n (%)
**Living situation**		
	Alone	384 (29.63)
	With (partner or family or friend)	912 (70.37)
**Education**		
	Low	474 (36.57)
	Intermediate	439 (33.87)
	Tertiary	383 (29.55)
**Work situation**		
	Working	332 (25.61)
	Not working	960 (74.07)
**Self-rated health**		
	Excellent	88 (6.79)
	Very good	245 (18.90)
	Good	548 (42.28)
	Fair	345 (26.62)
	Poor	70 (5.40)
**Self-rated quality of life**		
	Excellent	115 (8.87)
	Very good	335 (25.85)
	Good	574 (44.29)
	Fair	248 (19.14)
	Poor	24 (1.85)
**Current support need^a^**		
	No support	1073 (82.79)
	Domestic tasks	166 (12.81)
	Psychosocial support	88 (6.79)
	Personal care	45 (3.47)
	Medical care	30 (2.31)
**Support provider^a,b^**		
	Partner	95 (42.60)
	Child	62 (27.80)
	Family	13 (5.83)
	Friend	21 (9.42)
	Neighbor	13 (5.83)
	Professional	117 (52.47)
**Expected support need**		
	Highly unlikely	150 (11.57)
	Less likely than others	186 (14.35)
	Equally likely than others	577 (44.52)
	More likely than for others	114 (8.80)
	Highly likely	90 (6.94)
	Don’t know	179 (13.81)

^a^Multiple answers were allowed.

^b^Of those who reported to receive support (n=223).

### Survey Materials and Procedure

Participants were presented with a short (2.25 minutes) video animation that explained the concept of AAL [65]. Previous research has shown that animated content with spoken text works well to communicate complex health-related information [66]. For this video animation, a scenario was narrated with the persona Ben, an older adult, and his daughter and informal caregiver, Sophie. Personas and user scenarios are tools that are frequently used in participatory design activities to translate abstract ideas about the user into something more tangible [67]. Three example applications were included in the scenario: (1) smart home technology for activity monitoring and fall detection, (2) a reminder system for appointments and medications, and (3) a social service robot and a social companion robot. In addition to the video animation, participants viewed photos of market-ready AAL products: (1) Sensara activity monitoring [68], (2) Dayclocks reminder application [69], and (3) Zora, a social companion robot [70]. The photos contained a short description of the main features of the product. Two control questions were included to test the understanding of the presented material (“The video/pictures about AAL technology was/were clear to me”). Participants were also asked about their previous knowledge and experience with AAL technology.

After exposure to the video and photos, the participants were directed to the remaining items of the AAL acceptance survey. The survey concluded with questions about the sociodemographic background and participants’ self-rated subjective health and overall quality of life, received level of care, and anticipated need for care in the future.

### Measurements

Although some measurements were derived from validated scales, because of the lack of quantitative research in the field, a large part of the measurement was newly developed following the procedure described by DeVellis [71]. Topics from AAL literature and our qualitative user studies [60,61] were used as a starting point to create the initial pool of items. To test and improve the psychometric properties of the newly developed measurements and the overall survey structure, several pretests were conducted. First, the initial pool of items was evaluated for content validity, clarity, and redundancy with 4 senior researchers with expertise in AAL and psychometrics. After this first pretest, some items were removed and the others were rephrased. In the second pretest, the complete web-based survey instrument was presented to 3 older adults to evaluate the overall format (layout, structure, and length), test their ability to navigate through the web-based environment, and evaluate their comprehension of the survey items. Following the guidelines described by Willis [72], we conducted cognitive interviews using a combination of think-aloud and verbal probing techniques, while participants clicked through the survey. As a result, several problem areas were identified and the survey was adjusted accordingly.

We used a 5-point Likert scale as a response scale (1=strongly disagree; 5=strongly agree). For the attitude items, a 5-point semantic differential scale was used. *Don’t know* was included as a response option as AAL is a fairly new concept, and we suspected that some participants would not have a strong enough tendency to formulate an opinion [73]. The *don’t know* option was treated as missing values. Full information maximum likelihood (FIML) was used to deal with the missing values. FIML is considered a robust and state-of-the-art approach to handle missing data and is widely recommended in the methodological literature [74-77]. Table 2 gives a concise overview of the operationalization of the key variables included in the survey instrument. Multimedia Appendix 2 [43,44,47,55,56,78-90] shows the final list of items after validation.

**Table 2:** Measurements.

Variable name	Number of items in the survey	Example item
Intention to use AAL^a^	4	In the future, I intend to use AAL technology
Attitude toward using AAL	6	I (like/dislike) the idea of using AAL technology
Social norm	3	Most people whose opinion I value, would think positively about my use of AAL technology
Personal norm	3	I view myself as a user of technology for my health and well-being
Perceived behavioral control	4	Using AAL technology is entirely in my control
Safety	6	If I use AAL technology, I will feel safer in my home
Independence	4	If I use AAL technology, I can do things independently
Relief of family burden	6	My use of AAL technology will give my family members peace of mind
Loss of privacy	6	If I use AAL technology, I worry that my personal information might be shared with others without my permission
Loss of human touch	6	If I use AAL technology, I will get less personal attention
Caregiver influence	3	My caregivers would have a positive view on my use of AAL technology
Social stigma	4	If I use AAL technology, I am concerned that the technology will be visible to others
Human touch norm	4	I prefer personal care over care via AAL technology
Privacy norm	6	I think I have the right to control my personal information
Personal innovativeness	4	If I heard about a new information technology, I would look for ways to experiment with it
Self-efficacy	7	If I had problems relating to using AAL technology I know I could work them out
User control	3	I think that I will feel in control, when using AAL technology
Reliability	4	I think that AAL technology is reliable
Financial cost	3	I think that using AAL technology will be expensive

^a^AAL: ambient assisted living.

### Structural Equation Modeling

We used structural equation modeling (SEM) to validate the conceptual model.

The measurement model was validated in 2 stages. First, a pilot study was conducted among 320 older adults in the Netherlands. The hypothesized relationships between the latent variables and their indicator variables were explored using confirmatory factor analysis. Although this technique is labeled as confirmatory, it was used in an exploratory and iterative manner by paying attention to the posthoc modification indices [91]. By specifying the relationships between the latent variables and their indicator variables a priori, we employed a theory-driven approach rather than a data-driven approach to validate the measurements [92,93]. The measurement model was respecified with the main study sample (N=1296), leading to further refinement of the measurement model.

We used the Lavaan package version 0.5-23 [94] in R version 3.4.3 [95] to perform the analysis. Maximum likelihood estimation with FIML for missing data was used because the data were approximately normally distributed. The original measurement model proposed 19 distinct latent factors and 86 indicator variables. Indicators with poor standardized factor loading (<0.50) and low squared multiple correlation (SMC<0.40) were removed. To further evaluate the convergent validity of the measurement model, we assessed the McDonald hierarchical omega [96], Cronbach alpha [97], and the average variance extracted (AVE) for each latent variable. The threshold for the former 2 measurements was 0.70, and the recommended AVE value threshold was 0.50. Discriminant validity was examined using the heterotrait-monotrait (HTMT) ratio. If the HTMT value is <0.90, discriminant validity is established [98]. After validating the measurement model, the structural equation model was tested.

## Results

### Measurement Model

The fit measures of the original model were less than aspiration values. After the inspection of factor loadings and SMC values, several indicators were iteratively removed. This included the latent variable user control, as 2 of the 3 indicators loaded poorly on the latent construct. A minimum of 3 indicators are required to represent the latent variable [99]. The indicators of the latent variable privacy norm had low or just acceptable SMC values. As the variable showed relatively weak psychometric properties across the 2 independent samples, privacy norm was removed from the measurement model. One indicator (PSN03) with an SMC value less than the aspiration value was not excluded to meet the requirement of the 3 indicators to represent the latent variable. Another indicator less than the aspiration value (PI02) was included because it originated in a validated scale [78]. Upon inspection of the posthoc modification indexes, suggested residual correlations between the following indicator pairs were added: PSN03 and PI02, ATT02 and ATT03, ATT04 and ATT05, LP03 and LP05, LP03 and LP06, LP05 and LP06, and FB03 and FB05. After calculating the hierarchical omega, Cronbach alpha, and AVE values, it was decided to remove the latent variable social stigma from the measurement model because of a low AVE value (AVE=0.47) and overall weak psychometric properties across the 2 samples. Finally, HTMT values indicated that safety and independence should be considered as a single latent variable called *safe and independent living.*

The final measurement model consisted of 15 latent factors, 63 indicators, and 7 added residual correlations. The model showed acceptable-to-good fit for all fit measures (root mean square error of approximation [RMSEA]=0.04; standardized root mean square residual [SRMR]=0.05; comparative fit index [CFI]=0.93; and Tucker Lewis index [TLI]=0.92). Multimedia Appendix 3 displays the final list of indicators with intercept (FIML mean), indicator mean (values with listwise deletion), SD (values with listwise deletion), factor loadings, SMC, hierarchical omega, Cronbach alpha, and AVE.

### Descriptives

The indicator scores from the final measurement model were pooled into a composite score for each latent variable. Table 3 shows an overview of the composite mean, SD, and range for each latent variable.

**Table 3:** Composite mean and SD per latent variable.^a^

Latent variable	Mean (SD)	Minimum	Maximum
Intention to use AAL^b^	3.34 (0.73)	1	5
Attitude toward using AAL	3.73 (0.78)	1	5
Social norm	3.67 (0.57)	1	5
Personal norm	3.42 (0.75)	1	5
Perceived behavioral control	3.32 (0.71)	1	5
Safe and independent living	3.92 (0.52)	1	5
Relief of family burden	3.67 (0.65)	1	5
Loss of privacy	3.14 (0.87)	1	5
Loss of human touch	3.13 (0.83)	1	5
Caregiver influence	3.73 (0.56)	1	5
Human touch norm	3.97 (0.67)	1	5
Personal innovativeness	3.19 (0.78)	1	5
Self-efficacy	3.79 (0.60)	1	5
Reliability	3.26 (0.59)	1	5
Financial cost	3.81 (0.68)	1	5

^a^Single imputation with the Expectation Maximization method was used to handle the missing data for the composite scores and group comparison.

^b^AAL: ambient assisted living.

The overall intention to use AAL technology was moderately high in the sample (mean 3.34, SD 0.73). This means that, in general, older adults were relatively open to the idea of using AAL technologies in the future. Regarding the 3 age quotas, there was no significant difference in their use intention (F_2,1293_=2.89; *P*=.06). Similarly, we found no significant differences across different levels of subjective health (F_4,1291_=0.60; *P*=.66) and expected support needs (F_4,1112_=0.52; *P*=.72).

### Structural Equation Model

The hypothesized structural equation model showed good overall fit with the observed data: RMSEA=0.04, SRMR=0.06, CFI=0.93, and TLI=0.92. The model accounted for 69% of the variance in the intention to use AAL (*R*^2^=0.69). All but 2 of the hypothesized paths had significant standardized path coefficients at the *P*<.001 level.

**Figure 2:**
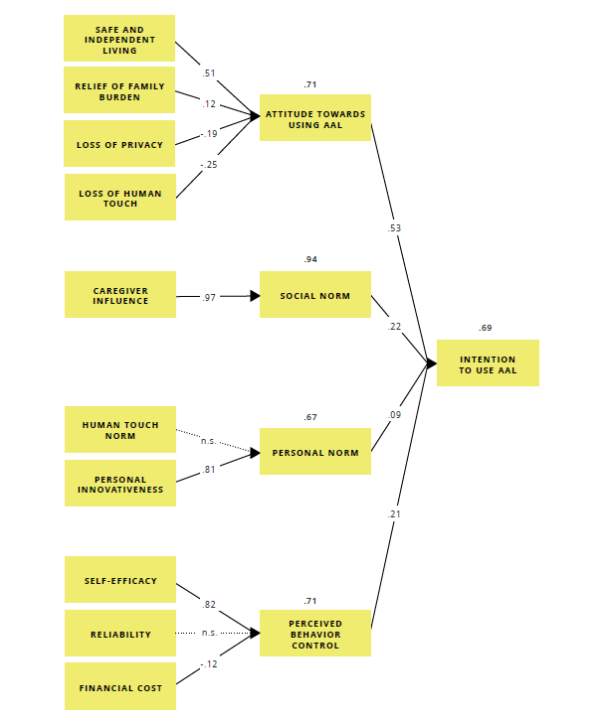
Structural equation model. Values adjacent to the single-headed arrows represent the standardized regression coefficients (*P*<.001). The dotted lines represent the nonsignificant paths. Values above the variable rectangles represent the variance explained in the latent variables.

Attitude toward using AAL, social norm, personal norm, and perceived behavior control significantly affected the intention to use AAL. Attitude was the most important influencer of intention (β=.53). Attitude toward using AAL was affected by older adults’ expectations about safe and independent living (β=.51), relief of family burden (β=.12), loss of privacy (β=–.19), and loss of human touch (β=−.25). Together, these variables explained 71% of the variance in attitude (*R*^2^=0.71). Social norm was strongly affected by caregiver influence (β=.97). Caregiver influence predicted 94% of the variance in social norm. The hypothesized influence of human touch norm on personal norm was not significant (*P*=.39), and personal innovativeness therefore remained to be the only significant predictor of personal norm (β=.81). Personal innovativeness explained 67% of the variance in personal norm. Self-efficacy (β=.81) and financial cost (β=−.12) remained to be the 2 predictors of perceived behavior control. Together, these variables explained 71% of the variance in the perceived behavior control. The expected influence of reliability was not significant (*P*=.68; Figure 2).

## Discussion

### Principal Findings

The aim of this study is to develop a statistically grounded understanding of the acceptance of AAL technology among older adults in the Netherlands. Specifically, this study aimed to compare the relative importance of different acceptance factors, their underlying relationships, and their explanatory power for the intention to use AAL technologies in the future.

The results of the web-based acceptance survey showed that the proposed model of AAL acceptance showed a good model fit for the observed data and explained 69% of the variance in intention to use. All hypothesized paths were significant, except for the path between human touch norm and personal norm and the path between reliability and perceived behavior control. Therefore, it can be concluded that our established theoretical model provides a valuable framework for understanding and explaining older adults’ acceptance in the early acceptance stage.

The overall intention to use AAL technology was moderately positive. This means that older adults are relatively open to the idea of using AAL technologies in the future. We found no difference in intention to use between age groups, people with different subjective health ratings, and different expected support needs. Although this might be somewhat surprising, this is in line with findings from Ziefle and Röcker [100], who found that age and subjective health status did not influence the willingness to use AAL technologies.

As expected, the intention to use AAL was predicted by attitude toward using AAL, social norm, personal norm, and perceived behavior control. Attitude was the most important predictor, followed by social norm and perceived behavior control. The results showed only a weak influence on personal norm. Ajzen [40] argues that “the relative importance of attitude, subjective norm, and perceived behavioral control in the prediction of intention is expected to vary across behaviors and situations.” From the results, we can conclude that in an early acceptance stage, in which people have no or limited experience with AAL technologies, the overall attitude toward using AAL is the most important influencer of intention to use. On the other hand, self-based standards and expectations regarding AAL use are only minor influencers of older adults’ intention to use.

Safe and independent living was the most important positive influencer of attitude, which in turn influenced intention to use. This is in line with previous AAL research [57,59,101] and our own qualitative user studies [60,61]. Older adults regarded the increased feeling of safety and the opportunity of independent living as a major advantage of AAL. We also found empirical evidence that promises of safety and autonomy are a valid trade-off for concerns about personal interaction and privacy, as suggested by earlier research [58,102]. Nevertheless, in line with previous studies [20,21,23-26], both concerns still substantially contributed to a negative attitude toward using AAL and should be considered when developing AAL applications. Earlier studies [58,101,103] suggested that older adults perceive AAL technologies as good tools for reducing the overall burden on caregivers. This was confirmed by the results of the AAL acceptance survey.

Previous research has suggested that the influence of caregivers, especially informal family caregivers, is important for the acceptance of AAL technologies [104-106]. Although we did not distinguish between formal and informal caregiver influence, the findings of the AAL acceptance survey indeed identified caregivers as crucial social referents for building social norm. Social norm, in turn, influenced use intentions. For future research, it would be interesting to explicitly distinguish between formal and informal caregiver influence.

In line with our qualitative user studies [60,61], older adults’ general willingness to try out new information technology positively contributed to their overall personal norm. However, the effect of personal norm on intention to use AAL was weak in the current sample. In contrast to our expectations, human touch norm had no significant influence on personal norm. An explanation for this finding may be that older adults preferred human care over care via AAL technology but could still identify as users of AAL.

Self-efficacy is a concept derived from social cognitive theory and is an essential determinant of human motivation and behavior [107,108]. Following previous research [57], it was hypothesized that self-efficacy would positively affect use intention via perceived behavior control. This hypothesized relationship was confirmed through the results of the AAL acceptance survey. Moreover, in line with previous research [57,101], expectations about high financial cost negatively contributed to perceived behavioral control. The hypothesized relationship between perceived behavior control and reliability was not significant. We suspect that with no or limited experience of AAL, users found it difficult to formulate specific and consistent expectations about the expected reliability of AAL. However, we believe that reliability will be considered in a later acceptance stage when users are actively interacting with the technology. Therefore, future research should consider these variables.

### Limitations

As in every study, there were some limitations to be considered. First, by using a web-based survey, we accepted that our sample had a bias toward older adults with internet connection and some technology skills. However, most older adults are active internet users [109,110]. Hence, the current sample remains to be largely representative of the older Dutch adult population. Second, participants’ responses were based on the provided study material and not on direct interaction with AAL technologies. This could have limited participants’ impressions of AAL. However, this fits the phase of early acceptance. In real-life situations, older adults will not necessarily try out a new technological device before forming their initial use intention. Previous research has shown that participants can form attitudes and expectations toward new and unfamiliar technologies without active use experience [32,111-113]. Finally, reaching an acceptable model fit in SEM does not imply that the hypothesized model is the only fitting model. Other equivalent or near-equivalent models may show equal or even better fit [93]. However, at this stage, the AAL field does not offer a rich theoretical discourse to inspire alternative models. Moreover, the measurement part of the model was cross-validated across 2 independent samples. In addition, the model was built on a strong and well-established psychological theory (TPB), a literature review, and several qualitative user studies.

### Future Research

To the best of our knowledge, this model is one of the first theory-driven quantitative frameworks for understanding AAL acceptance, which has been validated with a representative sample of the target population. However, this also means that this model is the first approximation to explain AAL acceptance. Further cross-validation and refinement is needed to ensure that this model remains stable and valid across different populations and cultural contexts. The established model focuses on early user acceptance and the initial intention to use AAL. Future research needs to implement longitudinal designs to explore later stages of acceptance when older adults start using the technology in their own home environment and attitudes, user needs, and intentions might change [34,35,114]. This study focused on older adults. Other important stakeholder groups include informal and formal caregivers. They can be primary users of AAL applications [115] and are important in signaling the older adults’ need for support and introducing AAL into the home care practice [7]. Hence, future research should further investigate caregivers’ perceptions of AAL.

For now, our insights into early acceptance among older adults can shape the further discourse and implementation of AAL.

### Conclusions

For the future success of AAL, it is vital to know if these technologies will fall on fertile ground and will be accepted by the intended users. In other words, will the policy vision of AAL as a solution to healthy and independent aging become reality from the perspective of older adult users? This study shows that Dutch older adults seem receptive to the idea of using AAL technology in the future. Being mindful of the acceptance factors will help developers make more informed design decisions before diffusing applications into the market.

Although the provided model focuses on AAL technologies, our insights on acceptance factors (eg, loss of privacy, loss of human touch, caregiver influence, financial cost) can also be valuable for the broader field of eHealth development and implementation.

## Appendix

Multimedia Appendix 1 Definition of the proposed belief constructs.

Multimedia Appendix 2 Refined measurements of the ambient assisted living acceptance survey.

Multimedia Appendix 3 Final measurement model intercept, mean, SD, standardized factor loadings, hierarchical omega, Cronbach alpha, and average variance extracted.

## Abbreviations

AAL: ambient assisted living
AVE: average variance extracted
CFI: comparative fit index
EU: European Union
FIML: full information maximum likelihood
HTMT: heterotrait-monotrait
ICT: information and communication technology
RMSEA: root mean square error of approximation
SEM: structural equation modeling
SMC: squared multiple correlation
SRMR: standardized root mean square residual
TAM: Technology Acceptance Model
TLI: Tucker Lewis index
TPB: theory of planned behavior
UTAUT: unified theory of acceptance and use of technology
