# Supplementary material for: Acceptance of Technologies for Aging in Place: A Conceptual Model
Source: J Med Internet Res. 2021 Mar 31;23(3):e22613. doi: 10.2196/22613 (PMC8047804; doi:10.2196/22613)
Supplement: Multimedia Appendix 1 [file jmir_v23i3e22613_app1.pdf]

Multimedia Appendix 1: Definition of the proposed belief constructs

Variable name	Definition
Safety	People's judgement of the likelihood that using AAL technology will lead to an enhanced sense of safety and security, faster detection and response to harmful situations in the home environment, and prevention of harmful situation in the home environment.
Independence	People's judgement of the likelihood that using AAL technology will enhance their independence and allow them to age in their own home environment.
Relief of family burden	People's judgement of the likelihood that using AAL technology will reduce family caregivers' emotional and physical burden.
Loss of privacy	People's judgement of the likelihood that using AAL technology will compromise their physical, psychological, social and informational privacy.
Loss of human touch	People's judgement of the likelihood that using AAL technology will decrease the human touch in care and face-to-face interaction.
Caregiver influence	People's perception that caregivers would encourage their use of AAL technology.
Social stigma	People's perception that important others will think they are old, frail and dependent when using AAL technology.
Human touch norm	People's judgement of the importance of the human touch in care and face-to-face interaction.
Privacy norm	People's judgements of the importance of privacy and data security when using AAL technology.
Personal innovativeness	People's willingness to try out any new information technology.
Self-efficacy	People's judgment of their capabilities to organize and execute courses of action required to use AAL technology.
User control	People's perceived sense of control over the interaction with AAL technology.
Reliability	People's belief that AAL technology will consistently operate properly.
Financial cost	People's belief that AAL technology use is associated with high financial expenses.
