# Supplementary material for: Acceptance of Technologies for Aging in Place: A Conceptual Model
Source: J Med Internet Res. 2021 Mar 31;23(3):e22613. doi: 10.2196/22613 (PMC8047804; doi:10.2196/22613)
Supplement: Multimedia Appendix 2 [file jmir_v23i3e22613_app2.pdf]

Multimedia Appendix 2: Refined measurements of the AAL acceptance survey

Item	Abbreviation	Source
**Intention to use AAL Technology**		2 items adapted from Ben Allouch et al., 2009
In the future, I plan to use AAL technology.	ITU01	
In the future, I expect to use AAL technology.	ITU02	
In the future, I intend to use AAL technology.	ITU03	
I would recommend other people to use AAL technology.	ITU04	
**Attitude towards using AAL Technology**		Adapted from Norman et al., 2000; Taylor & Todd, 1995; Ben Allouch et al. 2009
Using AAL technology is a (good/bad) idea.	ATT01	
Using AAL technology is (wise/foolish).	ATT02	
Using AAL technology is (valuable/worthless).	ATT03	
I (like/dislike) the idea of using AAL technology.	ATT04	
Using AAL technology is (pleasant/unpleasant).	ATT05	
Using AAL technology is (enjoyable/unenjoyable).	ATT06	
**Social norm**		Adapted from Mathieson, 1991; Taylor &Todd, 1995
Most people who influence me would have a positive opinion towards my use of AAL technology.	SN01	
Most people who are important to me would have a positive opinion towards my use of AAL technology.	SN02	
Most people whose opinion I value would think positively about my use of AAL technology.	SN03	
**Personal norm**		Adapted from Lee, Lee, & Lee, 2006; Sparks and Shepherd, 1992
I view myself as a user of technology for my health and well-being.	PSN01	
I think of myself as someone who is very interested in technology for health and well-being.	PSN02	
I am not the type of person oriented to use technology for my health and well-being .	PSN03 (-)	

**Perceived behavioral control**		Adapted from Taylor & Todd, 1995.
I would be able to use AAL technology.	PBC01	
Using AAL technology is entirely in my control.	PBC02	
I have the resources and opportunities it takes to make use of AAL technology.	PBC03	
I have the knowledge it takes to make use of AAL technology.	PBC04	
**Safe and independent living** *(merged into one construct after pilot study)*		3 items inspired by Roelands et al., 2002
Using AAL technology will give me a sense of security.	SAF01	
If I use AAL technology, I will feel safer in my home.	SAF02	
If I use AAL technology, accidents at home will be noticed immediately.	SAF03	
With the help of AAL technology, I will receive immediate help in case of emergencies.	SAF04	
Using AAL technology will allow me to age in my home environment.	IDEP01	
If I use AAL technology I can keep doing things on my own.	IDEP03	
If I use AAL technology I can do things independently.	IDEP04	
**Relief of family burden**		1 item inspired by Bedard et al., 2001
My use of AAL technology, will give my family members peace of mind.	FB01	
If I use AAL technology, my family members will be less concerned.	FB02	
If I use AAL technology, my family members will have more time for themselves.	FB03	
My use of AAL technology will relieve the burden on my family members.	FB05	
**Loss of privacy**		1 items inspired by Boise et al., 2013; 2 items inspired by Kirchbuchner et al., 2015
If I use AAL technology, I am concerned that others might use my personal information to harm me.	LP01	
If I use AAL technology, I worry that my personal information might be shared with others without my permission.	LP02	
If I use AAL technology, I worry to be constantly monitored.	LP03	

If I use AAL technology, I am concerned that my social interactions will be monitored.	LP04	
Using AAL technology will feel like an invasion into my personal space.	LP05	
If I use AAL technology, I am concerned that intimate situation will be monitored.	LP06	
**Loss of human touch**		1 item inspired by Roelands et al., 2002
If I use AAL technology, people will visit me less often.	LHT03	
If I use AAL technology, I will receive less personal care.	LHT04	
Using AAL technology, I will have get less personal attention.	LHT05	
Using AAL technology will replace human contact.	LHT06	
**Caregiver influence**		Adapted from Ajzen, 2006; Taylor and Todd, 1995
My caregivers would have a positive opinion towards my use of AAL technology.	CI01	
My caregivers would have a positive view on my use of AAL technology.	CI03	
My caregivers would value my use of AAL technology.	CI04	
**Human touch norm**		Adapted from Dabholkar, 1996; Phang et al., 2006
Human contact is more enjoyable than contact via AAL technology.	HTN01	
I Like interacting with humans more than interacting via AAL technology.	HTN02	
Personal attention is more important than attention via AAL technology	HTN03	
I prefer personal care over care via AAL technology.	HTN04	
**Personal innovativeness**		Adapted from Agarwal and Prasad, 1998
If I heard about a new information technology, I would look for ways to experiment with it.	PI01	
In general I am hesitant to try out new information technologies .	PI02 (-)	
Among my peers, I am usually the first to try out new information technologies.	PI03	
I like to experiment with new information technologies.	PI04	

**Self-efficacy**		3 items adapted from LaRose et al., 2012<br>2 items adapted from Meuter et al., 2003
I feel confident about using AAL technology.	SEF01	
I feel confident I know how to learn advanced skills related to using AAL technology.	SEF02	
I feel confident understanding terms/words relating to AAL technology.	SEF03	
I would avoid AAL technology because it is unfamiliar to me.	SEF06 (-)	
I hesitate to use AAL technology for fear of making mistakes I cannot correct .	SEF07 (-)	
**Reliability**		Adapted from Mcknight et al., 2011
I think that AAL technology is reliable	REL01	
I think that AAL technology will not fail me	REL02	
I think that AAL technology does not malfunction for me.	REL04	
**Financial cost**		2 items adapted from Luarn & Lin, 2005
It will cost a lot to use AAL technology	C01	
There are financial barriers to my use of AAL technology	C02	
I think that using AAL technology will be expensive.	C03	
