# Supplementary material for: Acceptance of Technologies for Aging in Place: A Conceptual Model
Source: J Med Internet Res. 2021 Mar 31;23(3):e22613. doi: 10.2196/22613 (PMC8047804; doi:10.2196/22613)
Supplement: Multimedia Appendix 3 [file jmir_v23i3e22613_app3.pdf]

Multimedia Appendix 3: Final measurement model intercept, mean, standard deviation, standardized factor loadings, hierarchical omega, cronbach's alpha, and average variance extracted.

Latent variable	Indicator	Intercept/ FIML mean	Listwise mean	Listwise SD	Factor loadings	SMC	α	ω^h	AVE
ITU	ITU01	3.31	3.39	0.85	.91	.82	.94	.87	.78
	ITU02	3.31	3.37	0.89	.90	.81			
	ITU03	3.26	3.32	0.86	.90	.82			
	ITU04	3.39	3.44	0.83	.80	.65			
ATT	ATT01	3.94	3.94	0.90	.84	.71	.93	.91	.67
	ATT02	4.03	4.03	0.83	.79	.63			
	ATT03	3.97	3.97	0.82	.81	.66			
	ATT04	3.46	3.46	1.05	.78	.61			
	ATT05	3.50	3.50	0.94	.84	.71			
	ATT06	3.49	3.49	0.89	.83	.69			
SN	SN01	3.51	3.57	0.76	.72	.52	.81	.89	.63
	SN02	3.68	3.74	0.68	.86	.73			
	SN03	3.71	3.77	0.65	.81	.65			
PSN	PSN01	3.32	3.32	0.91	.86	.73	.77	.79	.55
	PSN02	3.63	3.63	0.86	.80	.64			
	PSN03	3.31	3.31	0.97	.56	*.32*			
PBC	PBC01	3.68	3.73	0.77	.83	.69	.82	.78	.50
	PBC02	3.11	3.13	0.90	.70	.50			
	PBC03	3.09	3.10	0.98	.64	.41			
	PBC04	3.35	3.37	0.99	.69	.47			
SAF-IDEP	SAF01	3.81	3.82	0.71	.76	.58	.88	.91	.53
	SAF02	3.89	3.91	0.72	.77	.60			
	SAF03	4.06	4.08	0.60	.69	.47			
	SAF04	4.06	4.07	0.66	.64	.41			
	IDEP01	3.87	3.88	0.74	.75	.56			
	IDEP03	3.85	3.86	0.64	.70	.49			
	IDEP04	3.89	3.91	0.69	.75	.56			
FB	FB01	3.80	3.84	0.68	.87	.75	.85	.84	.57
	FB02	3.73	3.76	0.79	.83	.68			
	FB03	3.47	3.49	0.85	.62	*.39*			
	FB05	3.62	3.66	0.83	.73	.54			
LP	LP01	3.14	3.14	1.01	.86	.74	.93	.85	.63
	LP02	3.22	3.22	1.02	.91	.82			
	LP03	3.24	3.24	1.10	.74	.55			
	LP04	3.12	3.12	1.02	.90	.81			
	LP05	2.97	2.97	1.03	.64	.40			
	LP06	3.15	3.15	1.01	.70	.50			
LHT	LHT03	2.87	2.84	1.03	.69	.48	.87	.84	.61
	LHT04	3.35	3.36	0.98	.81	.65			
	LHT05	3.10	3.10	0.96	.84	.71			
	LHT06	3.20	3.20	1.06	.78	.61			

CI	CI01	3.63	3.69	0.68	.75	.56	.82	.92	.65
	CI03	3.72	3.77	0.67	.86	.74			
	CI04	3.74	3.78	0.68	.81	.65			
HTN	HTN01	4.19	4.19	0.73	.82	.67	.87	.88	.63
	HTN02	3.97	3.98	0.76	.79	.62			
	HTN03	3.99	4.00	0.76	.82	.68			
	HTN04	3.74	3.75	0.88	.76	.58			
PI	PI01	3.22	3.22	0.92	.84	.71	.84	.83	.57
	PI02	3.22	3.22	1.00	.62	*.38*			
	PI03	2.64	2.63	1.01	.75	.56			
	PI04	3.70	3.70	0.87	.82	.67			
SEF	SEF01	3.67	3.71	0.72	.80	.64	.82	.87	.50
	SEF02	4.08	4.10	0.67	.71	.50			
	SEF03	3.87	3.91	0.66	.75	.56			
	SEF06	3.71	3.73	0.87	.69	.48			
	SEF07	3.58	3.60	0.92	.63	.40			
REL	REL01	3.40	3.44	0.71	.84	.70	.84	.78	.60
	REL02	3.41	3.45	0.75	.76	.58			
	REL04	2.88	2.88	0.82	.74	.54			
C	C01	3.89	3.92	0.76	.85	.72	.86	.86	.68
	C02	3.58	3.57	0.98	.73	.54			
	C03	3.94	3.97	0.79	.92	.85			

ITU = intention to use AAL, ATT = attitude toward using AAL , SN = social norm, PSN = personal norm, PBC = perceived behavior control, SAF-IDEP = safe and independent living, FB = relief of family burden, LP = loss of privacy, LHT = loss of human touch, CI = caregiver influence, HTN = human touch norm, PI= personal innovativeness, SEF = self-efficacy, REL = reliability, C= financial cost
